# Mapping evidence on support networks and their impact on postnatal teenage mothers’ mental health in a global context: A scoping review

**DOI:** 10.4102/hsag.v31i0.3463

**Published:** 2026-10-05

**Authors:** Rinae Sikhwari, Fhumulani M. Mulaudzi, Robert Foromo

**Affiliations:** 1 Department of Nursing Sciences, Faculty of Health, University of Pretoria, Pretoria, South Africa

**Keywords:** support network, teenage mothers, mental health, postpartum depression, social support

## Abstract

**Background:**

Postnatal teenage mothers faced unique mental health challenges because of the combined effects of adolescence and early motherhood. While the importance of support networks was acknowledged, a comprehensive understanding of their influence across global and African contexts remains limited.

**Aim:**

This scoping review aimed to map existing research on support networks available to postnatal teenage mothers and explored their impact on mental health outcomes. The review included studies from healthcare and community settings across South Africa, Kenya, Ghana, United Kingdom, Indonesia, Turkey, Canada, Mozambique, Brazil, Iran, Jordan and Nairobi.

**Method:**

A systematic search identified 15 articles published between 2020 and 2025 from Web of Science, EBSCOhost, ProQuest, Google Scholar, and PubMed. Studies using qualitative, quantitative, and mixed-methods approaches were included after Preferred Reporting Items for Systematic Reviews and Meta-Analyses-guided screening of 1242 records, with data thematically synthesised.

**Results:**

Ten main themes emerged from thematic analysis of the included studies, namely lack of family support, lack of healthcare professional support, digital support, financial support, emotional support, physical support, absence and accessibility of partner or spousal support, lack of social support, mental conditions and support in developed settings. Family’s emotional or practical support protected against postpartum depression and anxiety, while partner involvement and financial stability significantly influenced outcomes. Digital support showed promise where formal services lacked accessibility.

**Conclusion:**

Integrated, adolescent-sensitive support networks proved critical for promoting mental health among postnatal teenage mothers. Addressing service gaps, social stigma and strengthening family or partner support remained essential for improving well-being in this vulnerable group.

**Contribution:**

This review mapped support network types and their mental health impacts, providing evidence for targeted policy development and clinical interventions tailored to teenage mothers’ unique needs across diverse global contexts.

## Introduction

Despite the challenges of teenage motherhood, many young mothers find a pathway to meaningful lives and bright futures after giving birth. Teenage motherhood is a challenging issue impacting hundreds of thousands of young teenage mothers throughout the world, particularly in resource-scarce countries with limited accessibility to different support networks (Muthelo et al. 2024). These young teenage mothers have enormous social, emotional and physical challenges as they become parents, while they are still progressing through their own development (Borg Xuereb, Borg Xuereb & Jomeen 2023). That sudden shift to motherhood leaves them significantly more vulnerable to mental health issues like postpartum depression, anxiety, low self-esteem and chronic stress (Noushad et al. 2021). This evidence highlights the interaction between teenagers’ vulnerability and motherhood, showing that insufficient support networks can easily affect their mental health. Establishing this understanding provides a basis for how the quality and availability of support networks play an important role in affecting the mental health of postnatal teenagers.

In the postnatal period, postnatal teenagers are supposed to get consistent care, guidance and support from their boyfriend, friends, family, community and the healthcare system to ease the transition to motherhood. These teenage mothers should have access to all support networks to safely guide their mental health. However, the situation is different, especially in low- and middle-income countries. Teenage mothers in Malawi experienced social stigma, which hindered their access to healthcare services, leading to feelings of isolation and shame (Tembo, Portsmouth & Burns 2023). Similar experiences are documented in Kenya, where limited parental support and social exclusion contribute to high rates of postpartum depression among adolescent mothers (Gebrekristos et al. 2025). A study in Ghana, which can be generalised to all countries, financial hardship and inadequate support networks increase vulnerability to anxiety and depression in teenage mothers, while dependence on exploitative relationships is a common coping mechanism (Nkansah, Ababio & Awuviry-Newton 2022). In South Africa, adolescent mothers contend with multifaceted challenges including familial disapproval, social isolation, poverty, lack of spousal support, healthcare worker stigma and limited educational opportunities, all of which negatively influence their mental health (Govender, Naidoo & Taylor 2020; Muthelo et al. 2024). Adding to this, studies from Iran indicate that unintended pregnancies and insufficient spousal support elevate the risks of postpartum depression and post-traumatic stress disorder (PTSD) among teenage mothers (Anbi et al. 2024). Contrastingly, in higher-income settings such as the United Kingdom, teenage mothers may have greater access to formal mental health services and structured support networks, yet institutional mistrust, stigma and past negative experiences often undermine effective service utilisation and psychological wellbeing. Taken together, these studies reveal that support networks should act as a protective measure for postnatal teenagers; the absence of support networks increases the risk of mental health conditions. This contrast between the expected provision of support and the reality of limited access underscores the need to examine how disrupted or inadequate support networks influence the mental health of postnatal teenage mothers.

Despite widespread recognition in public health guidelines such as ‘National integrated maternal and perinatal care guidelines for South Africa’ of the adverse consequences of teenage pregnancy, there remains a conspicuous shortage of treatments and interventions that place postnatal teenage mothers’ mental health at the centre. These interconnected issues not only make the experience of young motherhood more difficult but also have serious mental health issues. The gap addressed in this study lies in the tendency of existing research to examine postnatal mothers as a general population, with limited attention to teenagers. This scoping review seeks to close that gap by focusing specifically on postnatal teenage mothers, who represent a distinct group with unique vulnerabilities and support needs. Examining how support networks affect the mental health of these young mothers is important for recognising the positive and negative sides of the new life and experiences of teenage mothers postpartum (Nkansah et al. 2022). Leaving this gap unresolved risks continued cycles of untreated mental health problems, weakened mother-child relationships, disrupted education and broader social and health burdens.

A supportive, sympathetic and reliable support network among teenage mothers has been shown to be associated with lower rates of postpartum depression and other mental health challenges (Muthelo et al. 2024; Twintoh et al. 2025). The support network comprises peers, family, friends, healthcare personnel and community services, which is important in influencing the mental health of young teenage mothers. This implies that the evidence is now directing authorities to provide strategic and highly community-sensitive teenage programmes that are geared towards addressing psychological and social consequences of teenage motherhood (Abdul Rahim et al. 2024). This scoping review will adopt the five-step framework developed by Arksey and O’Malley (2005) and Fetzer (2025). Therefore, this study aims to explore the influence of support networks on the mental health outcomes of postnatal teenage mothers globally. This study’s aim is, therefore, to map the available evidence to clarify how support networks influence the mental health of postnatal teenage mothers across global and African contexts.

## Methodology

### Methods of search

This scoping review adhered to the five-step framework developed by Arksey and O’Malley (2005), further refined by Levac, Colquhoun and O’Brien (2010) and Fetzer (2025). By using this approach, the authors were able to incorporate evidence-based research, which was then summarised and prepared to be published. To enhance the rigour, transparency and reproducibility of the study selection process, the PRISMA (Preferred Reporting Items for Systematic Reviews and Meta-Analyses) 2020 flow diagram will be utilised in the study selection process. It followed a strict transparency procedure that allowed the search approach to be replicated, promoting the validity of the study’s conclusions (see Figure 1).

**FIGURE 1:**
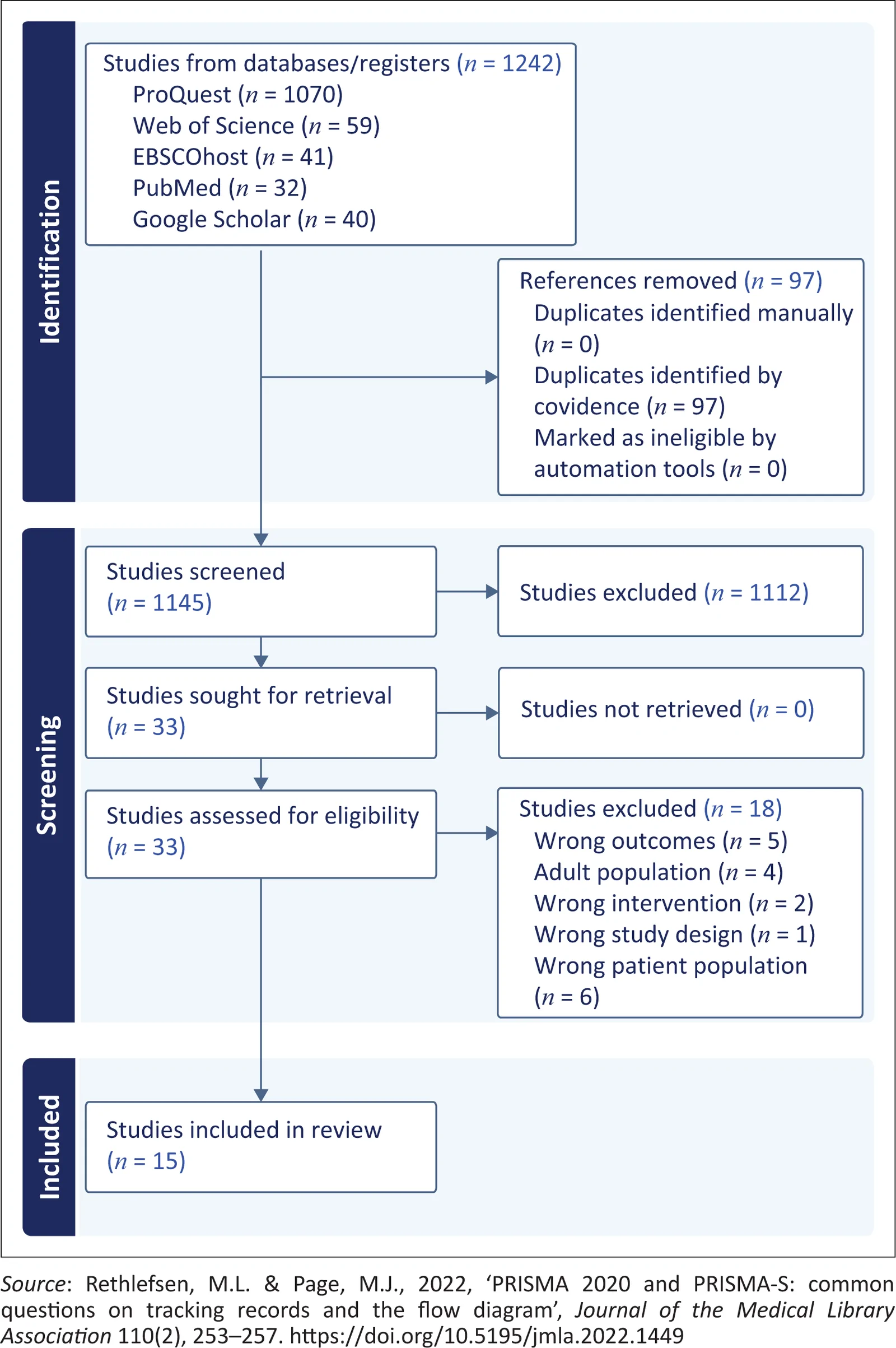
Preferred Reporting Items for Systematic reviews and Meta-Analyses study flowchart.

Scoping review question: *What types of support networks are available to postnatal teenage mothers, and how do these networks affect their mental health outcomes?*

### Information sources

The purpose of this search was to discover published studies, not forgetting the focus of the review. The authors collaborated with the university librarian to develop a search strategy for the review. Five main databases were used, and all the studies found were 1242, which were uploaded to the Covidence website for screening. The databases used to find the literature were Web of Science, EBSCOhost, ProQuest, Google Scholar and PubMed. Only English articles published between January 2020 and January 2025 were included in the search.

### Search strategy

To identify keywords from the research question, the following research string was used (Schiavenato & Chu 2021). Keywords and logical combinations utilised were: ‘Postnatal OR postpartum’ OR ‘after birth’ OR ‘post birth’ ‘Teenage mothers’ OR ‘Adolescent mothers’ OR ‘Young mothers’ OR ‘Teen mothers’ ‘Support networks’ OR ‘social support’ OR ‘peer support’ OR ‘family support’ OR ‘community support’ OR ‘healthcare support’ OR ‘emotional support’ OR ‘financial support’ OR ‘informal support systems’ OR ‘formal support systems’. ‘Mental health’ OR depression OR ‘postpartum depression’ OR anxiety OR stress OR ‘psychological wellbeing’ OR ‘emotional wellbeing’ OR ‘maternal mental health’. Studies were selected according to the inclusion and exclusion criteria. The inclusion and exclusion criteria used to guide the study selection process are presented in Table 1.

**TABLE 1:** The following were the requirements for inclusion, including exclusion criteria.

Inclusion	Exclusion
Studies involving postnatal teenage mothers aged 13–20 years worldwide.	Books
English language 2020–2025	Does not have full text
Qualitative, quantitative, mixed-method, cross-sectional, randomised controlled trial studies full text	Argumentative articles with no connection to the study
Anxiety, depression and poor mental well-being and positive outcome	-

### Inclusion and exclusion criteria

The researcher looked for studies published between 2020 and 2025 using Web of Science, EBSCOhost, ProQuest, Google Scholar and PubMed in accordance with PRISMA criteria. Studies that examined mental health outcomes in connection to support networks that focused on teenage mothers between the ages of 13 years and 20 years after giving birth were eligible. A total 1112 of article titles were shortlisted as irrelevant, while 15 article titles were screened as relevant. The selection process was visually summarised by the PRISMA flow diagram.

### Study selection and screening

Figure 1 illustrates the study selection and process. A total of 1242 records were identified across five databases. Most of these records were drawn from ProQuest (*n* = 1070), while smaller numbers were retrieved from Web of Science (*n* = 59), EBSCOhost (*n* = 41), PubMed (*n* = 32) and Google Scholar (*n* = 40). This distribution highlights ProQuest as the most substantial contributor to the pool of evidence. During the deduplication phase, 97 records were removed, leaving 1145 unique records. These were subjected to title and abstract screening, after which 1112 studies were excluded for not meeting the preliminary criteria. Consequently, 33 articles were advanced for full-text assessment. At the eligibility stage, the full texts of these 33 articles were carefully examined against the predefined inclusion and exclusion criteria. A total of 18 studies were excluded, with reasons clearly documented:

- Five studies focused on outcomes that were not relevant to the review.
- Four studies concentrated on adult populations, outside the scope of postnatal teenage mothers.
- Two studies examined pregnancy prevention and contraceptive interventions, which were outside the scope of this review.
- One study employed a study design that was not consistent with the review protocol.
- Six studies examined populations other than the target group.

Title reading and abstract reading were used to chart pertinent data from each study, using authorship, year, country, study design, population, type of support investigated and reported mental health outcomes. The key issue for the scoping review was used to determine the relevance of the abstracts. A standardised data extraction form was created. The primary reviewer carried out data extraction by hand, independently and with an accuracy cross-check.

### Data extraction

To ensure methodological rigour, three reviewers independently went through this process. Research topic clarity, ethical issues and the reliability of the results were important evaluation factors. Every study that was included satisfied the minimal requirements for quality. By mapping the identified literature and determining its similarities, differences and any gaps, the results were thematically synthesised. To address the study issue and provide a more comprehensive understanding of the relationship between support networks and postnatal teens’ mental health, the reviewers were able to synthesise the data from these 15 articles. The themes were arranged based on the kinds of support networks (financial, physical, professional, emotional, and digital) that were discussed and how they related to the mental health outcomes of teenage postpartum mothers. The Findings and Discussion section, which offers critical interpretations and cross-study comparisons, goes into additional detail on these issues. Data extracted from the included studies are summarised in Table 2, including the authors, year of publication, country, study design, population, support networks, and key findings.

**TABLE 2:** Summary of data from included studies.

Research title, authors, year and setting	Aim (A), design (D)	Population (P), sample size (SS)	Findings	Strengths (S), limitations (L) and link to the study (link)
1. Examining the associations between social support and postpartum depression symptoms among adolescent mothers in Nairobi, Kenya Gebrekristos et al. (2025) Kenya	A: To fully examine the various impacts of social support on teenage mothers D: Cross-sectional data of teenage mothers	P = Teenage mothers ≤ 1 year postpartum (aged 14–19 years) SS = 193	Postpartum depression symptoms were reported by a quarter of the participants, with a prevalence of 24.9%. When compared to teenage mothers with less supportive parents, those whose parents were very supportive of their daughters’ education were less likely to have depressive symptoms (ARR: 0.35, 95% CI: 0.20–0.61 for maternal support; ARR: 0.34, 95% CI: 0.13–0.90 for paternal support). Teenage mothers who confided in a close female friend were also less likely to experience depressive symptoms (ARR: 0.61; 95% CI: 0.37–0.99). Support network = Parental support	L = Cross-sectional design limits causality. Focused on urban sample, not generalisable to rural areas. S = Large sample size (*n* = 193), strong statistical analysis, direct measure of social support effects. Link = Highlights how parental and peer support reduces depression risk, reinforcing the need for strong emotional support networks among teenage mothers.
2. Psychosocial challenges and individual strategies for coping with mental stress among pregnant and postpartum adolescents in Nairobi informal settlements: a qualitative investigation Wainaina et al. (2021) Nairobi	A = To create a list of mental stressors experienced during pregnancy and early motherhood and understand their impacts. D = Qualitative interviews	P = Pregnant and teenage mothers aged 14–19 years SS = 30	The key psychosocial issues identified included being expelled from home by parents, financial difficulties, neglect from the partner, shame from family and community, shattered dreams, and living in poor conditions. Participants experienced emotions such as embarrassment, shame, hopelessness and, in severe cases, suicidal thoughts. Their coping mechanisms varied, with some opting for social isolation, while others connected with fellow teenagers or engaged in harmful behaviours like drug and alcohol use, as well as risky sexual relationships. The lack of preparation for early motherhood and inadequate psychosocial support heightened mental stress and depression risk, highlighting the need for a multifaceted approach to enhance the well-being of pregnant and teenagers’ mothers. Support network = family, financial and boyfriend support	S = Rich description of lived experiences; explored multiple stressors. L = Small sample; urban informal setting may not generalise. Link = Demonstrates how fragmented support networks increase risk of mental health breakdown in postpartum teens.
3. Maternal mental health and social support from online communities during pregnancy Jiang and Zhu (2022) UK	A = Investigate the impact of online community support on maternal mental health. D =Qualitative design	P = currently pregnant and gave birth within 1 year SS = 500	Users reported high levels of perceived social support (PSS) from online peer groups, comparable to support from family and friends in other studies. Linear regression analysis revealed that a one-point increase in PSS was linked to a 0.19-point decrease in prenatal depression (*p* < 0.1) and a 0.26-point decrease in postnatal depression (*p* < 0.01), representing reductions of 3% and 4.5%, respectively. Additionally, each one-point increase in PSS corresponded to a 14.49-gram increase in newborn weight (*p* < 0.01). Support network = Social support	S = Large sample; innovative focus on digital peer support. L = Only online population; PSS scores self-reported. Link = Validates digital platforms as viable support avenues, especially in low-resource rural settings.
4. The relationship between social support and maternal attachment of adolescent mothers and postpartum depression in Turkey Bulduk et al. (2025) Turkey	A = This study aimed to examine the relationship between social support, maternal attachment levels and postpartum depression among adolescent mothers in the at-risk group. D = Quantitative study design	P = Teenage mothers SS = 150 teenage mothers	The parameters influencing the mean Edinburgh postnatal depression scores were maternal age and the presence of assistance in infant care. The results showed that the mean scores of MSPSS sub-dimensions (significant other, family, friends), the total maternal attachment inventory scores, and the MAI scores increased, while the mean EPDS scores decreased. In addition, according to the regression analysis, both MAI (*t* = −5.206, *p* < 0.01) and MSPSS (*t* = −4.221, *p* < 0.01) have statistically significant effects on EPDS. This study recommends that adolescent mothers, as a vulnerable group, should receive support from social networks such as family, spouse, friends and neighbours during the postpartum period, with special consideration for the cultural context in which they live. More attention should be given to this group during the postpartum period. Multidimensional Scale of Perceived Social Support (MSPSS), Edinburgh Postnatal Depression Scale (EPDS), Maternal Attachment Inventory (MAI) Support = family, spouse, friends and neighbours’ support	S = The aim of study and the use measuring scale matches. L = This study focused on teenage mother without stating how long they are in postpartum. Link = This study emphasised with evidence from scale measurement that social support and maternal attachment influence postpartum depression.
5. Effectiveness of psychosocial support provided by midwives and family on PPD among first-time adolescent mothers at 3-month follow-up: randomised controlled trial Sangsawang et al. (2022) (Country = not stated)	A = To assess the impact of a midwife-family social support programme (MFPSS) on preventing postpartum depression (PPD) in first-time teenage mothers at 3 months postpartum. D = A randomised controlled trial	P = Teenage mothers SS = 40	At the last follow-up, all 20 participants (95.24%) remained in each group. Data analysis included 40 teenage mothers. After the intervention, the intervention group exhibited significantly lower mean EPDS scores compared to the control group at the post-test, 6-week, and 3-month postpartum follow-ups. Additionally, the rates and severity of PPD were reduced in the intervention group at these intervals. These findings suggest that psychosocial support interventions involving midwives and family members effectively prevent PPD in first-time teenage mothers, with benefits lasting up to 3 months postpartum. Support network = psychological support because of the prevalence of post-partum depression	S = Strong RCT design; family and midwife involvement sustained impact. L = Small sample; short-term follow-up (3 months). Link = Demonstrates structured family-healthcare interventions can reduce PPD in mothers.
6. Post-natal daily living experiences of unmarried teenage mothers: A phenomenological study in Enyan Denkyira, Ghana Ababio et al. (2022) Ghana	A = This study examined the daily post-natal experiences of teenage mothers in Enyan Denkyira. D = Phenomenological research design	P = Teenage mothers SS = 15	The findings revealed that financial challenges led to hunger and nutritional deficiencies among the mothers, negatively affecting their health. Additionally, most teenage mothers had dropped out of school and lacked financial, family and vocational support and skills, hindering their ability to earn an income and boost their self-esteem.	S = Highlights long-term effects of unsupported teen parenting. L = Very small sample; limited diversity in participants. Link = Reinforces the economic and psychosocial challenges from lack of financial and vocational support.
7. Post-natal daily living experiences of unmarried teenage mothers: A phenomenological study in Enyan Denkyira, Ghana Muthelo et al. (2024) South Africa	A = To examine the mental health and difficulties faced by teens who are pregnant and parenting was the goal of the study. D = Qualitative descriptive, exploratory and phenomenological design.	P = pregnant and parenting teenagers 18 years or older SS = 22	The results show that teens in rural locations who are pregnant and raising children face several mental health issues, including depression. These difficulties stem from societal issues such as stigmatisation, a lack of family and friend support, and parenting responsibilities that lead to subpar academic performance or school dropouts. Teens who were expecting voiced their worries about the loss of support from their spouses because of leaving their partners. Support network = peers, spousal and family support	S = Rural-specific findings; cultural sensitivity. L = No male partner or father insights; regionally focused. L = Strong match with the study because it shows urgent need for family and community engagement.
8. Antenatal and postpartum depression: Prevalence and associated risk factors among adolescents in KwaZulu-Natal, South Africa Govender et al. (2020) KZN	A = The purpose of this study was to explore the various viewpoints of a heterogeneous group of medical professionals who work in a district hospital in Ugu, KwaZulu-Natal, South Africa, providing care and interacting with teenage mothers and pregnant teenagers. D = Descriptive qualitative study	P = Healthcare professionals who offer care to teenagers who are pregnant or parenting were gathered from the HIV or AIDS, STIs and TB (HAST) programme as well as the maternity, prenatal, paediatrics, psychology, dietetics, physiotherapy and social work departments. S = 33	The medical professionals in uGu district agreed that teenage pregnancy is a serious concern. Teenage mothers’ reproductive and sexual health after giving birth, they also believed, was not prioritised. According to the healthcare experts, the issues faced by teenagers who are pregnant, or parenting include school dropout, financial hardship, relationship disintegration, desertion, stigmatisation, challenges with parenting and childrearing, and issues related to both physical and mental health. Teenage mothers and pregnant teens receiving judgmental counselling Support network = provided by healthcare professional	S = Multisectoral healthcare practitioners’ perspectives; policy implications. L = Did not include teenagers’ viewpoints. Link = Highlights service delivery gaps for teens in health systems.
9. Postpartum depression and PTSD and resultant risk factors among teenage mothers: A community-based study Anbi et al. (2024) Iran	A = To investigate the prevalence of depression, distress and post-traumatic stress disorder (PTSD) following childbirth and their resultant risk factors among teenage mothers. D = Cross-sectional study	P = Teenage girls aged 11–19 years, 8 weeks after giving birth SS = 167	The findings showed that PTSD was predicted by factors such as unintended pregnancies, inadequate income, lack of spousal support and baby restlessness. In conclusion. Our results highlight the importance of identifying adolescent moms who have risk factors for PTSD and postpartum depression. Essential interventions to address the mental health issues faced by adolescent moms include teaching fathers to assist their spouses, offering family planning counselling to minimise undesired pregnancies, and making sure high-risk individuals are referred promptly. PTSD Support network = spousal and financial support	S = Used validated tools (EPDS, PCL-5, PDM). And identified key social and economic risk factors (lack of spousal support, inadequate income, unwanted pregnancy, infant restlessness). L = Focused on one city (Tabriz), so findings may not represent all teenage mothers in Iran or globally. Link = Focused on one city (Tabriz), so findings may not represent all teenage mothers in Iran or globally.
10. Integrating human-centred design into the development of an intervention to improve the mental wellbeing of young women in the perinatal period: The Catalyst project Taylor Salisbury et al. (2021) Mozambique	A = Collaborate with stakeholders in Mozambique to develop evidence-based interventions and delivery strategies that align with the needs and priorities of all involved parties. D = Qualitative, human-centred design approach	P = Young pregnant women and new mothers (ages 16–24), their families, community leaders, service providers, and government representatives. SS = The study included 65 participants: 23 young pregnant women or new mothers, 12 family members, 19 service providers, and 11 Ministry of Health staff.	Participants expressed considerable uncertainty regarding their living conditions, financial situations, education, social support and understanding of pregnancy and parenting’s effects. Family and community support emerged as essential for this group. The Mama Feliz (Happy Mama) programme was created with stakeholders to enhance knowledge about pregnancy, childbirth and child development, while also fostering positive relationships, problem-solving and parenting skills. Additionally, family sessions focus on broader cultural and gender issues that affect teenagers’ maternal well-being. Support network = social support	S = Participatory model; culturally grounded. L = Wide age group; focus not solely on mental health outcomes. Link = Reinforces importance of building community-based and educational support interventions.
11. Association between Islamic religiosity, social support, marriage satisfaction and postpartum depression in teenage mothers in West Java, Indonesia: A cross-sectional study Nurbaeti, Lestari and Syafii (2023) Indonesia	A = To investigate correlations between Islamic religiosity, social support, marital satisfaction and PPD in teenage mothers. D = Cross-sectional design	P = Teenage postpartum mothers SS = 203	According to the study, postpartum depression symptoms were present in 35.96% of teenage mothers. Marital satisfaction (*p* = 0.002), education level (*p* = 0.012), family income (*p* = 0.003), number of children (*p* = 0.044) and birth weight (*p* = 0.0001) were significant factors linked to this condition. In contrast, there was no significant correlation found between postpartum depression and religiosity (*p* = 0.788) or social support (*p* = 0.688). Multiple logistic regression analysis revealed that the most significant factors were number of children (*p* = 0.026; OR = 3.68; 95% CI: 1.173–11.547), family income (*p* = 0.001; OR = 0.326; 95% CI: 0.165–0.644), and marital satisfaction (*p* = 0.001; OR = 0.365; 95% CI: 0.197–0.676). Support network = financial support And spousal satisfaction	S = Large sample; multivariate statistical analysis; diverse factors analysed. L = Social support measured broadly; cultural specifics may limit global comparison. Link = Create a crucial contrast: That in developed settings like Indonesia with family-structured support, social support alone may not predict mental health outcomes. Financial and marital satisfaction were the problem-causing postpartum depression.
12. Young mothers’ use of and experiences with mental healthcare services in Ontario, Canada: A qualitative descriptive study Jack et al. (2022) Canada	A = To outline the experiences of young mothers who got mental health services during the perinatal period and to pinpoint the qualities of the services and providers that affected their choice to seek mental health services D = Qualitative component of a sequential explanatory mixed-methods study	P = (≤ 21 years) who met diagnostic criteria for at least one psychiatric disorder, and who were ≥ 2 months postpartum SS = 29 young mothers.	Many young mothers shared that what they needed most was simply someone to talk to whether a trusted friend, family member or a caring professional. For those who considered reaching out to formal mental health services, the decision was often clouded by fear and past hurt. Some had been judged, others had felt pushed aside in their own care or were anxious about being prescribed medication or triggering unwanted involvement from child protection services. These experiences made support feel risky rather than safe. Mothers also reflected on how the attitudes and approach of healthcare providers and organisations affected whether they felt comfortable enough to seek help or stay engaged in care. Support network = family support and healthcare support	S = Rich personal narratives; clear exploration of service-related barriers; applied to a clinical diagnosis group. L = Focused on a high-income setting; excludes teens without a psychiatric diagnosis. Link = Highlights emotional and relational aspects of professional support, showing how trust, fear and service quality influence mental health help-seeking in young mothers.
13. Post-natal daily living experiences of unmarried teenage mothers: A phenomenological study in Enyan Denkyira, Ghana Nkansah et al. (2022) Ghana	A = Exploring effective coping and intervention strategies used by unmarried teenage mothers. D = Phenomenological design	P = 15 unmarried teenage mothers, 10 church leaders, and two District Assembly officials SS = 27	According to the survey, to make ends meet, young mothers in Enyan Denkyira continued to work in manual labour-intensive jobs, including washing other people’s clothes and collecting water for building projects. It was clear that women were forced to rely on men once their other means of income failed and the help from supporters stopped coming, making them lifelong victims of unplanned pregnancies and early motherhood. Support network = financial support	S = Included multiple perspectives (teen mothers, religious leaders, government officials); rich description of survival-based coping; context-specific insight. L = Small sample; limited focus on direct mental health outcomes. Link = Shows how weak or lost support pushes teen mothers into risky situations, linking to stress and emotional hardship.
14. Mental health issues in adolescent mothers and young adult mothers: The Brazilian context Carvalho et al. (2025) Brazill	A = To analyse the mental health of Brazilian adolescent mothers who use the Unified Health System D = Multicentre design	P = Teenage mothers (10–19 years old) SS = 583	Some level of depression (61.9%) and anxiety (63.8%) was present in the adolescents. These symptoms were associated with a lack of family support (*p*-value < 0.001), the loss of school friends (*p*-value < 0.001), the diagnosis of postpartum depression (*p*-value < 0.001), and the diagnosis of depression or anxiety before or during pregnancy (*p*-value < 0.001). 15.9% of the adolescents diagnosed with mental health conditions were being treated. The main barriers identified were difficulties in access to psychological support, lack of interest and lack of encouragement. Support network = family support and peers’ support	S = Large sample size, increased transferability and reliability L = This study does not clarify which category teenage mothers fall into; whether they are all postpartum or had passed postpartum. Link = This study supports and links to the study by linking family and peers’ support as one of the most significant supports which are influencing depression and anxiety.
15. Informative title: Effects of social support, parenting stress and self-efficacy on postpartum depression among adolescent mothers in Jordan Mohammad et al. (2021) Jordan	A = This study investigated the effects of social support, parenting stress and maternal self-efficacy on postpartum depression among adolescent mothers in Jordan. D = Cross-sectional design	P = Teenagers aged less than 20 years; 6–8 weeks postpartum S = 200	The findings showed that postpartum depression was likely to be present in 28.5% of teenage moms. Compared to mothers who did not report these stressors, mothers who reported high levels of social support, high parenting stress, low self-efficacy, financial stress and marital conflict scored considerably higher on the EPDS. EPDS	S = The study became more specific about the population size and collecting data from a large number of participants, which ensures credibility and reliability. L = Cross-sectional design prevents causal conclusions. Link = This study investigates the effects of social support and parenting stress, which influence the mental health of these teenagers, for example, EPDS.

Note: Please see full reference list of this article: Sikhwari, R., Mulaudzi, F.M. & Foromo, R., 2026, ‘Mapping evidence on support networks and their impact on postnatal teenage mothers’ mental health in a global context: A scoping review’, *Health SA Gesondheid* 31(0), a3463. https://doi.org/10.4102/hsag.v31i0.3463 for more information.

OR, odds ratio; ARR, adjusted risk ratio; CI, confidence interval; RCT, randomised controlled trial; HIV, human immunodeficiency virus; STIs, sexually transmitted infections; AIDS, acquired immunodeficiency syndrome; TB, tuberculosis; UK, United Kingdom; KZN, KwaZulu-Natal.

#### Synthesis

The results of the included studies were compiled and interpreted using a narrative synthesis approach (Ade-Ojo et al. 2022).

## Review findings

Fifteen studies conducted between 2020 and 2025 met inclusion criteria, spanning diverse global settings including South Africa (*n* = 2), Kenya (*n* = 1), Ghana (*n* = 2), United Kingdom (*n* = 1), Indonesia (*n* = 1), Turkey (*n* = 1), Canada (*n* = 1), Mozambique (*n* = 1), Brazil (*n* = 1), Iran (*n* = 1), Jordan (*n* = 1), Nairobi (*n* = 1) and one randomised controlled trial without a specified country (*n* = 1).

These represented low-, middle- and high-income contexts. Study designs included qualitative (phenomenological, descriptive, human-centred), quantitative (cross-sectional) and mixed-methods approaches.

In all, 10 main themes emerged from thematic analysis of the included studies: Lack of family support, lack of healthcare professional support, digital support, financial support, emotional support, physical support, absence and accessibility of partner or spousal support, lack of social support, mental conditions and support in developed settings. The summary of included studies is attached in a separate document.

The quality of support networks and the mental health outcomes of teenage postpartum mothers were consistently linked, according to the comprehensive research. Nine main themes emerged from the thematic analysis of the 15 included studies: 10 main themes emerged from thematic analysis of the included studies namely: Lack of family support, lack of healthcare professional support, digital support, financial support, emotional support, physical support, absence of partner support, lack of social support, mental conditions and support in developed settings. A summary of each theme area and how it affects mental health is found below. These networks, which include social, family-related and community resources, all support young mothers’ general well-being as they deal with the difficulties of motherhood and mental health issues

### Lack of family support

The importance of family support, encompassing emotional, practical and occasionally financial assistance, is a common finding throughout the studies. Eleven studies showed how affected teenage mothers were as a result of a lack of family support. In Kenya, teenage mothers with high parental support, especially those whose parents encouraged their education, exhibited significantly lower rates of postpartum depression than those with no parental support (Gebrekristos et al. 2025). Similarly, confiding in close female friends was associated with reduced depressive symptoms, highlighting peer support’s role as a protective factor.

Contrasting these positive effects, several studies explain that the absence of family or peer support exacerbates symptoms of depression, social isolation and low self-esteem among teenage mothers. For instance, in rural South Africa, teenage mothers lacking emotional support from friends and family were found to experience heightened sadness and social disengagement (Muthelo et al. 2024). In Brazil, a large sample of teenage mothers showed a strong association between depression, anxiety and lack of family or peer support, with only a fraction accessing psychological treatment because of barriers such as poor service availability and lack of encouragement (Carvalho et al. 2025). Collectively, these findings emphasise that family-oriented support networks are a critical first line of defence against poor mental health outcomes in postnatal teenage mothers.

### Lack of healthcare professional support

Studies acknowledge notable shortcomings in professional healthcare support available to teenage mothers, particularly in low-resource settings. In Limpopo province, South Africa, pregnant and parenting teenagers face limited access to mental health services, which are further challenged by stigmatisation and a lack of expertise among healthcare workers to address this demographic’s specific needs (Muthelo et al. 2024). Similarly, multi-disciplinary healthcare professionals in KwaZulu-Natal reported that teenage mothers’ postnatal mental health is often deprioritised because of systemic gaps and limited teenage mothers-focused interventions (Govender et al. 2020). On the other hand, a randomised controlled trial in Thailand demonstrated that a midwife-family psychosocial support programme significantly lowered postpartum depression scores and severity up to 3 months postpartum in first-time teenage mothers, highlighting the effectiveness of structured, combined health and family interventions (Sangsawang et al. 2022). In Canada, the attitudes and approach of healthcare providers directly influenced young mothers’ willingness to seek and remain engaged with mental health services. Negative experiences involving judgment or fear of child protective services reduced help-seeking, while supportive and empathetic care facilitated engagement (Jack et al. 2022). These studies collectively suggest that while gaps remain, professional healthcare support, when accessible and sensitive, can play a vital role in improving teenage mothers’ mental health, whereas a lack of appropriate support may negatively impact their mental health.

### Digital support

Few (*n* = 2) studies explored digital avenues of support; however, the available evidence presents digital platforms as promising emerging sources of social support. In the United Kingdom, pregnant and postpartum teenage mothers who participated in online peer support forums reported high levels of PSS comparable to traditional family or friend support, which corresponded to lower prenatal and postnatal depression scores. Additionally, a positive association with infant birth weight was observed (Jiang & Zhu 2022). Complementing digital supports, community-based participatory programmes like Mozambique’s ‘Mama Feliz’ fostered knowledge-sharing, strengthened social relationships and addressed cultural and gender issues affecting teenage mothers’ maternal well-being, indicating that community and digital supports can work synergistically to fulfil practical and emotional needs (Taylor Salisbury et al. 2021). The findings suggest digital support could expand accessibility to vital psychosocial assistance, especially in resource-limited or stigmatised environments.

### Financial support

Financial support or the lack thereof emerged as a pervasive factor influencing teenage mothers’ mental health internationally. In Ghana, limited financial resources compelled teenage mothers to engage in labour-intensive survival work or become dependent on potentially exploitative relationships because of insufficient family or vocational support (Nkansah et al. 2022). Similarly, hunger, school dropout and absence of income-generating opportunities compounded mental health challenges for Ghanaian teenage mothers (Ababio et al. 2022). In Indonesia, postpartum depression was significantly associated with low household income, number of children and marital dissatisfaction, rather than broad social support, underscoring socioeconomic determinants within a setting with relatively better structured social supports (Nurbaeti et al. 2023). Financial stress was also a significant predictor of postpartum depression among Jordanian teenagers, alongside parenting stress and marital conflict (Mohammad et al. 2021). A study in Iran reported that unintended pregnancies, inadequate income and lack of spousal support predicted higher rates of postpartum depression and PTSD, highlighting the intersection of economic and relational risk factors (Anbi et al. 2024). These findings converge on financial hardship as a central source of psychological stress and vulnerability for postnatal teenage mothers.

### Emotional support

Emotional support from family, peers, and partners plays a central role in mitigating mental health issues. Teen mothers who have trusted confidants report fewer depressive symptoms and better coping when emotionally supported (Gebrekristos et al. 2025). A lack of emotional support is correlated with feelings of sadness and isolation in rural South African communities (Muthelo et al. 2024). Emotional safety within both informal networks and professional interactions influences whether young mothers seek and continuously engage with mental health services. Judgmental or dismissive healthcare experiences foster fear and avoidance, whereas empathetic and supportive care promotes utilisation (Jack et al. 2022).

### Physical support

Physical or practical support, such as help with infant care and household chores, is cited as reducing the considerable physical and emotional load of early motherhood. Teenage mothers receiving such assistance report diminished feelings of exhaustion, irritability and stress in critical postpartum periods marked by hormonal adjustment and sleep deprivation (Gebrekristos et al. 2025; Muthelo et al. 2024). Similar observations were made among rural South African teenage mothers, where community members providing physical support contributed to a reduced caregiving burden and improved emotional well-being (Muthelo et al. 2024). These findings highlight that tangible assistance complements emotional and financial support in safeguarding psychological health among teenage mothers.

### Absence and accessibility of partner or spousal support

While partly covered under family or partner support (*n* = 15), the nature and impact often differ, especially regarding financial assistance, emotional engagement or desertion. Spousal or partner support emerges as a distinct and influential dimension shaping mental health outcomes. Lack of partner support, including financial neglect and emotional absence, frequently predicts increased postpartum depression symptoms and psychological distress. In Jordan, marital conflict alongside low spousal support correlated with higher postpartum depression scores (Mohammad et al. 2021). South African teenage mothers often experience abandonment by partners, exacerbating emotional distress and contributing to reduced social and economic resources (Muthelo et al. 2024). Similarly, Kenyan studies also indicate that a lack of partner support compounds loneliness and depression risk (Gebrekristos et al. 2025). This theme underscores the necessity to consider partner dynamics separately, as the presence or absence of supportive intimate relationships critically impacts mental health trajectories for postnatal teenage mothers.

### Lack of social support

Several studies emphasise the role of broad social support systems, including peer groups, community programmes and institutional interventions, in influencing postnatal teenage mothers’ mental health. Community-based participatory programmes in Mozambique integrate educational and psychosocial elements to enhance social cohesion and coping skills among teenage mothers (Taylor Salisbury et al. 2021). Similarly, peer support programmes reported by Jiang and Zhu (2022) highlight how connection within social networks reduces isolation and depressive symptoms. These forms of support extend beyond emotional comfort, providing information on how these teenage mothers could manage and fight against mental health challenges.

### Mental health conditions

The studies consistently report a high prevalence of postpartum mental health challenges among teenage mothers globally. Postpartum depression was the most frequently assessed condition, with prevalence rates ranging from approximately 25% to over 60% in different settings, reflecting significant vulnerability in this group (Carvalho et al. 2025; Gebrekristos et al. 2025; Govender et al. 2020). Anxiety symptoms, stress and social isolation were also commonly documented, often co-occurring with depressive symptoms (Muthelo et al. 2024; Nkansah et al. 2022). Some studies highlighted PTSD linked to unintended pregnancies and inadequate partner support (Ababio et al. 2022; Anbi et al. 2024; Bulduk et al. 2025).

The severity of these mental health problems varied depending on access to support networks, socioeconomic status and cultural stigma. Understanding the spectrum and magnitude of mental health outcomes is critical as it frames the urgency for practical support and intervention strategies, which are explored in subsequent themes.

### Support in developed settings

In developed countries, social support may not be a good predictor of mental health outcomes when formal mental health systems are well established. This is because organised professional services are available in these settings, which may make people less dependent on informal support systems, such as family and friends Nurbaeti et al. (2023). Say that there is no statistically significant link between teenage mothers’ postpartum depression and how they feel about social support in Indonesia. Instead, factors like birth weight, number of children, household income and marital happiness were more closely linked to mental health outcomes.

This means that social support may not have as much of an effect on mental health in structured institutions as in countries with less or no access to personal and socioeconomic factors.

This argument is further continued and supported by Jack et al. (2022), who found that even though there were official mental health treatments available, teenage mothers often chose not to use them. This was because they had bad experiences with mental health professionals in the past, were afraid of being judged, and didn’t trust the healthcare system. These institutional and psychological barriers made official and informal support systems less effective. These results show that having support networks, whether they are family or institutional, does not always lead to better mental health outcomes in high-income settings. Instead, the effectiveness of support depends on how good it is, how easily it can be accessed, and the willingness and ability of teenage mothers to engage with or utilise it. This shows how important it is to not only provide services but also to build trust and deal with the hidden emotional and social barriers that can make structured mental health systems less effective.

## Discussion

This scoping review aimed to explore the influence of support networks on the mental health outcomes of postnatal teenage mothers globally, with a particular focus on young mothers aged 13–20 years and the types of support from family, healthcare professionals, digital, financial, physical and spousal support that shape their mental wellness. The central research question was framed as: *What types of support networks are available to postnatal teenage mothers, and how do these networks influence their mental health?*

This scoping review set out to clarify how different support networks impact the mental health of postnatal teenage mothers, a population facing unique vulnerabilities because of their dual transition through teenage motherhood and early motherhood. The synthesis of evidence reveals that support is not a monolithic concept but rather a multidimensional construct encompassing emotional, financial, practical, professional, spousal and increasingly digital domains. Recognising this complexity is essential because it highlights that addressing teenage mothers’ mental health requires interventions that transcend conventional healthcare provision and engage multiple social systems.

The prominence of family support in influencing mental health underscores the foundational role that familial relationships play in shaping teenage mothers’ wellbeing during motherhood, making it one of the most influential and widely reported determinants of mental health (Muthelo et al. 2024). This suggests that interventions prioritising family engagement could reduce common mental health challenges such as postpartum depression. However, the documented variability and at times absence of family support, particularly in stigmatising environments, reflect broader social inequalities and cultural dynamics that may reinforce teenage mothers’ disadvantage. The implications are clear: Supportive family contexts offer protective buffers; however, where these are lacking, alternative or supplementary support networks, such as healthcare professional support, become critical (Tembo, Portsmouth & Burns 2025).

Healthcare professional support emerged as a double-edged factor, critical when accessible and sensitive, but often insufficient or inaccessible. The findings reflect systemic gaps in teenage mothers’ specific mental health services, highlighting how insufficient provider training, stigmatisation, and fragmented care compromise mental health support opportunities (Govender et al. 2020; Sangsawang et al. 2022). This highlights the importance of policy and training initiatives that focus on teenage mothers-friendly, stigma-reducing practices within maternal care systems. Furthermore, the demonstrated positive outcomes of integrated midwife and family support for mental health outcomes suggest that multisectoral collaboration can effectively fill existing service gaps (Sangsawang et al. 2022). This gap highlights the need for alternative support options, including digital and community-based services.

The growing recognition of digital support platforms is particularly noteworthy. Given resource constraints and social stigma surrounding teenage pregnancy in many settings, digital peer support and community-based programmes may represent scalable, culturally adaptable methods to provide psychosocial support (Jiang & Zhu 2022). These findings align with global trends in teenage health promotion, underscoring the need to integrate digital technologies into comprehensive care. Digital support cannot fully address material struggles, making financial stability the next support network.

Financial insecurity consistently emerges as a pivotal determinant of mental health. The strong linkage between economic hardship and psychological distress in teenage mothers exposes structural inequities that transcend individual-level interventions (Nkansah et al. 2022; Twintoh et al. 2025). This indicates that mental health outcomes cannot be fully addressed without concurrent socioeconomic support strategies aiming to reduce poverty-induced stressors (Ababio et al. 2022). Thus, holistic approaches must incorporate economic empowerment, education continuation and vocational training as essential components of mental health promotion for teenage mothers (Moberg, Skogens & Schön 2023).

Emotional and physical supports are tightly intertwined with mental health resilience. The qualitative evidence reveals that emotional safety, whether from family, peers or partners, is necessary not only to shield against postpartum depression but also to encourage utilisation of formal mental health services (Bulduk et al. 2025). Moreover, practical help in alleviating the burden of childcare allows teenage mothers to conserve their emotional and physical resources, thereby fostering better mental health outcomes. The role of emotional and physical support is closely tied to intimate relationships, bringing us to the role of spousal or partner support.

Spousal or partner support, as a distinct theme, adds another layer of complexity. The detrimental effects of partner absence or neglect amplify teenage mothers’ vulnerability, emphasising the relational dimension of postpartum mental health (Anbi et al. 2024). Interventions must thus account for relationship dynamics, promoting partner involvement where possible and addressing abandonment or conflict that exacerbate psychological distress.

### Implications and recommendations

The recommendations emphasise the need for targeted research to identify the specific forms of support that teenage mothers benefit from most, alongside long-term studies to track their mental health outcomes. Community-based projects, such as mentorship programmes that connect experienced and new mothers, are crucial for strengthening support networks. Educational initiatives on coping skills and mental health awareness can encourage engagement.

Additionally, training programmes for family members and community support workers should focus on developing communication and empathy skills to better support young mothers. Establishing peer support groups can foster a sense of community and reduce feelings of loneliness. To enhance support for teenage mothers, it’s crucial to provide recommendations aimed at healthcare providers and improving health access. Healthcare providers should receive training on the unique challenges faced by adolescent mothers, focusing on sensitivity, effective communication and the provision of comprehensive reproductive and mental health services. This can help create a supportive environment where young mothers feel comfortable seeking care. Overall, implementing these strategies and conducting further research can significantly enhance the mental well-being of teenage mothers and support the healthy development of their children.

## Conclusion

This scoping review mapped how support networks influence postnatal teenage mothers’ mental health globally. Multidimensional support (family, financial, healthcare and digital) proved essential for reducing depression or anxiety risks. Addressing gaps through integrated, adolescent-sensitive interventions represents the key way forward.

## References

[CIT0001] Ababio, M.O., Awuviry-Newton, K., Nkansah, J.O. & Abekah-Carter, K., 2022, ‘Post-natal daily living experiences of unmarried teenage mothers: A phenomenological study in Enyan Denkyira, Ghana’, *Journal of Education, Society and Behavioural Science* 35(5), 46–56. 10.9734/jesbs/2022/v35i530425

[CIT0002] Abdul Rahim, K., Egglestone, N.J., Tsagareli, I.G., Usmani, W., Meherali, S. & Lassi, Z.S., 2024, ‘Mental health outcomes beyond the post-partum period among adolescent mothers: A systematic review and meta-analysis’, *Health Psychology and Behavioral Medicine* 12(1), 2305741. 10.1080/21642850.2024.2305741 PMC10836488 38313449

[CIT0003] Ade-Ojo, G.O., Markowski, M., Essex, R., Stiell, M. & Jameson, J., 2022, ‘A systematic scoping review and textual narrative synthesis of physical and mixed-reality simulation in pre-service teacher training’, *Journal of Computer Assisted Learning* 38(3), 861–874. 10.1111/jcal.12653

[CIT0004] Anbi, A.A., Jahdi, N.S., Ranjbar, F. & Hakimi, S., 2024, ‘Postpartum depression, and post-traumatic stress disorder and resultant risk factors among teenage mothers: A community-based study’, *Discover Public Health* 21(1), 149. 10.1186/s12982-024-00282-y

[CIT0005] Arksey, H. & O’Malley, L., 2005, ‘Scoping studies: Towards a methodological framework’, *International Journal of Social Research Methodology* 8(1), 19–32. 10.1080/1364557032000119616

[CIT0006] Borg Xuereb, C., Borg Xuereb, R. & Jomeen, J., 2023, ‘Adolescent pregnancy and early parenting’, in R. Borg Xuereb & J. Jomeen (eds.), *Perspectives on midwifery and parenthood*, pp. 95–105, Cham, Springer. 10.1007/978-3-031-17285-4_8

[CIT0007] Bulduk, M., Kurt Can, E., Can, V. & Ayşin, N., 2025, ‘The relationship between social support and maternal attachment of adolescent mothers and postpartum depression in Turkey’, *BMC Pregnancy and Childbirth* 25(1), 603. 10.1186/s12884-025-07703-z PMC12101017 40405065

[CIT0008] Carvalho, A.F.d., Guaranha, D.D.F.K., Marmett, B., Reis, J.M., Rosa, B.S.D., Souza, C.L.E.D. et al., 2025, ‘A multicenter study on the mental health of Brazilian adolescent mothers, 2024’, *Epidemiologia e Serviços de Saúde* 34, e20240226. 10.1590/s2237-96222025v34e20240226.pt PMC12435885 40929498

[CIT0009] Fetzer, S.J., 2025, ‘Considering a scoping review’, *Journal of PeriAnesthesia Nursing* 40(4), 1075. 10.1016/j.jopan.2025.05.004 40750134

[CIT0010] Gebrekristos, L.T., Ajayi, A.I., Groves, A.K. & Kabiru, C.W., 2025, ‘Examining the associations between social support and postpartum depression symptoms among adolescent mothers in Nairobi, Kenya’, *Reproductive Health* 22(1), 11. 10.1186/s12978-025-01943-0 PMC11783768 39885595

[CIT0011] Govender, D., Naidoo, S. & Taylor, M., 2020, ‘“I have to provide for another life emotionally, physically and financially”: Understanding pregnancy, motherhood and the future aspirations of adolescent mothers in KwaZulu-Natal South, Africa’, *BMC Pregnancy Childbirth* 20(1), 620. 10.1186/s12884-020-03319-7 PMC7557067 33054778

[CIT0012] Jack, S.M., Duku, E., Whitty, H., Van Lieshout, R.J., Niccols, A., Georgiades, K. et al., 2022, ‘Young mothers’ use of and experiences with mental health care services in Ontario, Canada: A qualitative descriptive study’, *BMC Women’s Health* 22(1), 214. 10.1186/s12905-022-01804-z PMC9172978 35672725

[CIT0013] Jiang, L. & Zhu, Z., 2022, ‘Maternal mental health and social support from online communities during pregnancy’, *Health and Social Care in the Community* 30(6), e6332–e6344. 10.1111/hsc.14075. PMC10092248 36256486

[CIT0014] Levac, D., Colquhoun, H. & O’Brien, K.K., 2010, ‘Scoping studies: Advancing the methodology’, *Implementation Science* 5(1), 69. 10.1186/1748-5908-5-69 PMC2954944 20854677

[CIT0015] Moberg, J., Skogens, L. & Schön, U.-K., 2023, ‘Young people’s recovery processes from mental health problems – A scoping review’, *Child and Adolescent Mental Health* 28(3), 393–407. 10.1111/camh.12594 35960215

[CIT0016] Mohammad, K.I., Sabbah, H., Aldalaykeh, M., Albashtawy, M., Abuobead, K.Z., Creedy, D. et al., 2021, ‘Informative title: Effects of social support, parenting stress and self-efficacy on postpartum depression among adolescent mothers in Jordan’, *Journal of Clinical Nursing* 30(23–24), 3456–3465. 10.1111/jocn.15846 33988270

[CIT0017] Muthelo, L., Mbombi, M.O., Mphekgwana, P., Mabila, L.N., Dhau, I., Tlouyamma, J. et al., 2024, ‘Exploring mental health problems and support needs among pregnant and parenting teenagers in rural areas of Limpopo, South Africa’, *BMC Women’s Health* 24(1), 236. 10.1186/s12905-024-03040-z PMC11015670 38614989

[CIT0018] Nkansah, J.O., Ababio, M.O. & Awuviry-Newton, K., 2022, ‘Life-changing coping mechanisms and resilience strategies of post-natal unmarried teenage mothers in the central region, Ghana’, *Journal of Education, Society and Behavioural Science* 35(7), 13–26. 10.9734/jesbs/2022/v35i730434

[CIT0019] Noushad, S., Ahmed, S., Ansari, B., Mustafa, U.-H., Saleem, Y. & Hazrat, H., 2021, ‘Physiological biomarkers of chronic stress: A systematic review’, *International Journal of Health Sciences* 15(5), 46–59. PMC8434839 34548863

[CIT0020] Nurbaeti, I., Lestari, K.B. & Syafii, M., 2023, ‘Association between Islamic religiosity, social support, marriage satisfaction, and postpartum depression in teenage mothers in West Java, Indonesia: A cross-sectional study’, *Belitung Nursing Journal* 94(4), 313–321. 10.33546/bnj.2661 PMC10461155 37645571

[CIT0021] Rethlefsen, M.L. & Page, M.J., 2022, ‘PRISMA 2020 and PRISMA-S: common questions on tracking records and the flow diagram’, *Journal of the Medical Library Association* 110(2), 253–257. 10.5195/jmla.2022.1449 PMC9014944 35440907

[CIT0022] Sangsawang, B., Deoisres, W., Hengudomsub, P. & Sangsawang, N., 2022, ‘Effectiveness of psychosocial support provided by midwives and family on preventing postpartum depression among first-time adolescent mothers at 3-month follow-up: A randomised controlled trial’, *Journal of Clinical Nursing* 31(5–6), 689–702. 10.1111/jocn.15928 34196048

[CIT0023] Schiavenato, M. & Chu, F., 2021, ‘PICO: What it is and what it is not’, *Nurse Education in Practice* 56, 103194. 10.1016/j.nepr.2021.103194 34534728

[CIT0024] Taylor Salisbury, T., Atmore, K.H., Nhambongo, I., Mintade, M., Massinga, L., Spencer, J. et al., 2021, ‘Integrating human-centred design into the development of an intervention to improve the mental wellbeing of young women in the perinatal period: The Catalyst project’, *BMC Pregnancy and Childbirth* 21(1), 183. 10.1186/s12884-021-03675-y PMC7936480 33673826

[CIT0025] Tembo, C., Portsmouth, L. & Burns, S., 2023, ‘Postnatal depression and its social-cultural influences among adolescent mothers: A cross sectional study’, *PLOS Global Public Health* 3(6), e0002025. 10.1371/journal.pgph.0002025 PMC10289363 37352145

[CIT0026] Tembo, C., Portsmouth, L. & Burns, S., 2025, ‘“They organized women who advised me”: Adolescents’ perceptions of social and cultural influences on mental health during the postnatal period in Malawi: Qualitative study’, *PLOS Mental Health* 2(2), e0000044. 10.1371/journal.pmen.0000044 PMC12798599 41661728

[CIT0027] Twintoh, R.F., Amu, H., Kruh, V., Kissah-Korsah, K., Esia-Donkoh, K. & Darteh, E.K.M., 2025, ‘Postnatal experiences of teenage mothers in selected communities in central region, Ghana: A phenomenological study’, *BMC Public Health* 25(1), 327. 10.1186/s12889-025-21376-5 PMC11770965 39865258

[CIT0028] Wainaina, C.W., Sidze, E.M., Maina, B.W., Badillo-Amberg, I., Anyango, H.O. et al., 2021, ‘Psychosocial challenges and individual strategies for coping with mental stress among pregnant and postpartum adolescents in Nairobi informal settlements: a qualitative investigation’, *BMC Pregnancy and Childbirth* 21(1), 661. 10.1186/s12884-021-04128-2 PMC8480022 34583684

